# Factors associated with eating performance in older adults with dementia in long-term care facilities: a cross-sectional study

**DOI:** 10.1186/s12877-021-02315-6

**Published:** 2021-06-15

**Authors:** Dukyoo Jung, Jennie C. De Gagne, Hyesoon Lee, Minkyung Lee

**Affiliations:** 1 College of Nursing, Ewha Womans University, Seoul, Korea; 2 Duke University School of Nursing, Durham, NC USA; 3 Department of Thoracic Surgery, Mount Sinai Hospital, New York, USA

**Keywords:** Activities of daily living, Dementia, Dependency, Eating, Long-term care

## Abstract

**Background:**

The purpose of this study was to investigate factors influencing eating performance in older adults with dementia (OAWDs) in long-term care (LTC) facilities.

**Methods:**

This cross-sectional study examined risk factors for compromised eating performance by comparing both independent and dependent older adults with dementia. The study participants were 117 OAWDs in LTC facilities in South Korea. Measurements included (a) general characteristics, (b) activities of daily living (ADL) including eating performance, (c) cognitive function, (d) physical capability, (e) grip strength, (f) Behavioral Psychological Symptoms of Dementia (BPSD), and (g) depression. Data were analyzed by the percentage, mean and standard deviation, Chi-square test, t-test, and logistic regression.

**Results:**

The eating independent group had more comorbidities than the dependent group (*t* = 2.793, *p* < .006); had significantly higher cognition (*t* = 4.108, *p* < .001) and physical capability (*t* = 5.258, *p* < .001); and had stronger grip strength (*t* = 2.887, *p* = .005). Comorbidities and physical capability were determinants for independent eating performance (Odds Ratio [OR] = 1.969, *p* = .014; OR = 1.324, *p* < .001).

**Conclusions:**

It is suggested that maintaining physical capability should be encouraged to support independent eating performance by OAWDs in LTC facilities. The results of this study could serve as a basis for developing function-focused care to maintain the residual eating performance of OAWDs in Korean LTC facilities. This is a subject area that has not been fully explored.

## Background

Cognitive function is important for maintaining independence in older adults. Deterioration in cognitive function has been associated with greater risk of developing disability in basic activities of daily living (ADLs) [1]. Dementia in older adults is a progressive disease of cognitive impairment that causes a decline in the ability to perform ADLs [2] and can result in death. Most residents in long-term care (LTC) facilities receive a combination of physical, mental, and social support to help them perform ADLs [3] as the need for assistance and support with ADLs varies according to sex, age, and education level [4, 5]. Older adults with decreased physical capability, increased diagnosis of chronic diseases, or a lower than optimal weight may experience reduced ability to perform ADLs and have an increased need for assistance [2, 5].

Eating performance is defined as the function of getting food into the mouth [6], and for older adults residing in LTC facilities, it is an important ADL for ensuring sufficient dietary intake for the maintenance of healthy body weight, and is used as a quality measure for long-term care facilities [7]. Compromised eating performance can lead to malnutrition, infection, and poor quality of life [8]. A study conducted on residents of LTC facilities found that 80% of individuals with advanced dementia experience disturbances in chewing and swallowing, 48% have issues with poor intake, and 11% experience weight loss [9]. Other studies have found that residents with mild-to-moderate dementia are at a higher risk for malnutrition [10], and that residents with advanced dementia have significantly lower eating performance [11].

A study investigating eating performance among older adults with dementia (OAWDs) residing in LTC facilities in South Korea found that majority of the participants needed direct care workers to help with meals; 24.2% of these participants were severely dependent (i.e., needed total meal assistance from direct care workers) [12]. In a previous study, significant factors that have been shown to influence independent eating performance were considered according to a social ecological model that identified multi-domains related to eating performance, including (a) intrapersonal (resident characteristics), (b) interpersonal (caregiver characteristics), (c) environmental (facility or social culture contexts), and (d) policy (staffing) [6, 11, 13]. In the intrapersonal domain particularly, cognitive status, physical function, comorbidity, and psychological distress have been observed to affect eating behaviors of OAWDs [6, 14–16].

Reports regarding the association between factors of independent eating and OAWDs have presented varying conclusions: For instance, a study by Liu and colleagues [6] determined that physical performance and cognitive status are significant factors of independent eating performance in older adults with moderate to severe dementia [6], yet a study by Slaughter and colleagues [15] stated that eating disabilities may not arise due to compromised cognitive function but rather from comorbidities during middle-stage dementia [15]. Furthermore, while dementia-related behaviors such as behavioral and psychological symptoms of dementia (BPSD) should be taken into account when considering independent eating behavior in OAWDs, there is no previous study to verify the effects of BPSD on independent eating behaviors. The purpose of this study, therefore, was to evaluate the association between intrapersonal factors affecting independent eating performance in OAWDs living in LTC facilities in South Korea.

## Methods

### Study design, setting, and population

This descriptive cross-sectional study included residents aged ≥65 years with a diagnosis of dementia. Participants were selected through convenience sampling from multiple LTC facilities in which nurses work. Of the 130 residents who were recruited, 13 were excluded owing to deteriorated conditions, communication difficulties, or refusal to participate. The data were collected between April 9 and May 26, 2018 from five cities in South Korea.

### Data collection procedure

We selected seven LTC facilities, based in five cities in South Korea, for data collection and obtained approval from the directors of facilities after we explained the study proposal and data collection procedures. Lists of all eligible residents were provided by the directors of facilities. We initially approached these residents to evaluate their capacity for making decisions, and we obtained written informed consent from those residents who demonstrated an acceptable decision-making capacity. Consent for residents who could not comprehend the purpose of the study and/or make an informed decision to participate was provided by health care proxies such as family members. Approximately 10% of participants personally provided informed consent, while consent for 90% of the participants was provided by their health care proxies. This study was approved by the Institutional Review Board of the Ewha Womans University (No. 148–14).

Before data collection, four research assistants and nine nurses from the chosen LTC facilities completed a 1-h training session on the administration of the assessment instruments. At the end of the training session, the degree of inter-rater reliability was measured, and the result was a kappa value of 0.82, indicating acceptable inter-rater agreement.

### Measures

The questionnaire collected information on participants’ demographic characteristics, ADLs, cognitive function, physical capability, grip strength, BPSD, and depression. Demographic characteristics included age, sex, marital status, length of stay (in months), comorbidities, and level of LTC service. The level of LTC service was determined by assessing participants’ diseases or symptoms, visual/hearing ability, ADLs, cognition level, behavioral changes, and need for nursing care/rehabilitation/medical devices. LTC service levels ranged from level one, where participants required help in all aspects of daily life, to level five, where participants had dementia with limited functional decline [17]. In this study, levels one and two were defined as severe status, level three as moderate status, and levels four and five as mild status. The nurses in the LTC facilities assessed the following general characteristics, which were measured by minute observations in daily life: ADLs, BPSD, and depression. Cognitive function, physical capability, and grip strength were assessed by two researchers and four trained research assistants. We obtained permissions to use all survey instruments from authors or publisher.

#### ADLs

The Korean Activities of Daily Living (K-ADL), a tool developed by Won et al. [18] based on the Katz Index [19], was used to assess the participants’ level of ADLs. K-ADL comprises seven questions on dressing, washing the face, bathing, feeding, transferring, toileting, and continence. The scores range from 7 to 21 points with lower scores indicating higher independence. In the study that developed the K-ADL, the Cronbach’s α was 0.937 [18] compared with 0.91 in the present study.

#### Eating performance

Eating performance was assessed using the item “feeding” in the K-ADL [18]. This single item (“does the participant feed oneself without help?”) is rated on a scale of 1 (“completely independent to feed self”), 2 (“partially dependent and some assistance needed”), or 3 (“completely dependent and needs to be fed”). In this study, 1 point indicated independent eating performance, while 2 and 3 points indicated dependent eating performance.

#### Cognitive function

Cognitive function was measured using the Korean Mini Mental State Examination (K-MMSE) [20]. The K-MMSE is the Korean standardized version of the MMSE developed by Folstein et al. [21]. The total possible score is 30 points, with higher scores indicating higher cognitive function: 20 ≤ MMSE< 24 indicates mild impairment, 10 ≤ MMSE< 20 indicates moderate impairment, and 0 ≤ MMSE< 10 indicates severe impairment [20]. The Cronbach’s α for MMSE was 0.86 [21], and the sensitivity of the K-MMSE ranged between 0.70 and 0.83 [20]. The Cronbach’s α in this study was 0.90.

#### Physical capability

The Korean version of the Physical Capability Scale (PCS), translated by Jung et al. [22] and based on the PCS developed by Resnick et al. [23], was used to measure participants’ physical capability. This tool comprises 16 questions assessing the extent to which the participant can follow the examiner’s instructions, and it tests the ability and range of the joints in the arms and legs. One point is scored if the participant can perform the instruction, and 0 points are scored if they cannot. A higher score indicates higher physical capability. When the tool was first developed, the Cronbach’s α was 0.83, and it was 0.90 in our study.

#### Grip strength

A grip strength dynamometer (Model KS-301; Lavisen Co. Ltd., Namyangju, Korea) was used to measure the participants’ grip strength in kilogram-force (kgf). The participant grabbed the instrument with their predominant hand. Grip strength was measured in the sitting position with the engaged arm fully extended and hanging down; however, for patients who could not sit, grip strength was measured in the supine position with the engaged arm extended. Grip strength was measured twice using the same method, and the higher of the two measurements was selected. If there was a substantial difference between the two measurements, the grip strength test was repeated. The range of normal grip strength in the 65–69 age group is 28.2–44.0 kgf (men) and 15.4–27.2 kgf (women) and 21.3–35.1 kgf (men) and 14.7–24.5 kgf (women) in the 70–99 age group (Lavisen Co. Ltd., Namyangju, Korea).

#### BPSD

To assess the participants’ level of BPSD, we used the Neuropsychiatric Inventory-Questionnaire (NPI-Q) originally developed by Kaufer et al. [24] and translated to Korean [25]. For each participant, a nurse indicated the presence of 12 psychiatric symptoms (delusion, hallucination, agitation/aggression, depression/dysphoria, anxiety, euphoria/elation, apathy/indifference, disinhibition, irritability/lability, aberrant motor behavior, night-time behavior, and appetite/eating change) over the prior 4 weeks as present (“yes”) or not present (“no”). If “yes” was indicated, the symptom severity was assessed using a 3-point scale: 1 = mild, 2 = moderate, and 3 = severe. The total possible NPI-Q score ranged from 0 to 36, with a higher score indicating severe BPSD. The reliability of the NPI-Q in the previous study was Cronbach’s α = 0.75 [26], and in this study it was Cronbach’s α = 0.71.

#### Depression

The Cornell Scale for Depression in Dementia (CSDD), developed by Alexopoulos et al. [27] and translated into Korean by Kim [28], was used to assess participants’ depression. For each participant, a trained nurse was asked to describe 19 depressive behaviors observed during the previous week by rating them using a 3-point scale: 0 = absent, 1 = mild or intermittent, 2 = severe, or N/A = unable to evaluate. The total possible score ranged from 0 to 38 points, with a score ≥ 8 points indicating depression. In the study that developed the Korean CSDD [28], the Cronbach’s α was 0.84, and in our study, it was 0.77.

### Statistical analysis

Data were analyzed using SPSS Version 21.0 (Armonk, NY: IBM Corp.). Participant characteristics were analyzed using descriptive statistics (frequency, percentage, mean, and standard deviation). General characteristics were expressed as frequency, percentage, mean, and standard deviation. The level of ADLs, cognitive function, physical capability, grip strength, BPSD, and depression between eating performance (“independent” vs. “dependent”) groups were compared using Chi-squared (χ^2^) and *t*-tests. Logistic regression analysis was performed to identify predictors of independent eating performance. Variables that were potential predictors of independent eating performance (comorbidities, cognitive function, physical capability, and grip strength) were selected from the conceptual framework based on the literature review and results of the bivariate analysis performed in this study.

## Results

### Participant characteristics

The average age of participants was 86.2 years (SD = 6.21; range, 65–103), and the majority were female and widowed (86.3%). The mean length of stay in LTC facilities was approximately 2 years and 4 months, and the average number of comorbidities was 2.65 (SD = 1.10; range, 1–6). Level of LTC service was severe for 23.9% of the participants, moderate for 41.3%, and mild for 35.7%. Approximately one-third of the participants (31.6%) were fully or partially dependent for eating and needed assistance. On average, the participants had moderately impaired cognition, with a mean MMSE score of 12.13 (SD = 5.30); 9.4% of the participants exhibited mild impairment, 54.7% exhibited moderate impairment, and 35.9% exhibited severe impairment. The mean participant ADL score was 13.97 (SD = 4.57), PCS was 11.46 (SD = 4.20), and grip strength was 7.30 (SD = 6.41), indicating dependence on help for daily activities, with low levels of physical capability and weak grip strength. Participants also had lower levels of BPSD and depression, with a mean NPI-Q score of 5.74 (SD = 4.78) and CSDD score of 5.12 (SD = 4.41) (Table 1).

**Table 1:** Comparison of participant characteristics in the eating-dependent and eating-independent groups (*N* = 117)

Characteristics	Categories	Independent (*n* = 80)	Dependent (*n* = 37)	Total (*N* = 117)	χ^2^ or t	*p*
		*n* (%), Mean (*SD*)				
Age (year)		85.85 (5.99)	87.05 (6.68)	86.23 (6.21)	−0.974	.332
Sex	Male	10 (12.50)	6 (16.22)	16 (13.63)	0.296	.773
	Female	70 (87.50)	31 (83.78)	101 (86.32)		
Marital status	Married	8 (10.00)	4 (10.81)	12 (10.26)	1.918	.481
	Bereaved	68 (85.00)	33 (89.19)	101 (86.32)		
	Others	4 (5.00)	0 (0.00)	4 (3.42)		
Level of LTC service	Severe	15 (18.75)	13 (35.13)	28 (23.93)	3.964	.142
	Moderate	34 (42.50)	14 (37.84)	48 (41.03)		
	Mild	31 (38.75)	10 (27.03)	41 (35.04)		
Length of stay (months)		29.76 (25.77)	27.38 (24.60)	29.01 (25.32)	0.472	.638
Comorbidities		2.84 (1.13)	2.24 (0.92)	2.65 (1.10)	2.793	.006
Cognitive function		13.41 (4.66)	9.35 (5.59)	12.13 (5.30)	4.108	<.001
Physical capability		12.89 (2.87)	8.38 (4.96)	11.46 (4.20)	6.196	<.001
Grip strength (kg)		8.40 (6.89)	4.91 (4.43)	7.30 (6**.**41)	2.819	.006
BPSD		5.90 (4.82)	5.38 (4.74)	5.74 (4.78)	0.551	.583
Depression		5.34 (4.53)	4.65 (4.17)	5.12 (4.41)	0.783	.435

*LTC* Long-term Care, *MMSE* Mini Mental State Examination, *PCS* Physical Capability Scale, *BPSD* Behavioral and Psychological Symptoms of Dementia, *NPI-Q* Neuropsychiatric Inventory-Questionnaire, *CSDD* Cornell Scale for Depression in Dementia

### Differences in participant characteristics between the eating dependent and eating independent groups

No statistical significance was detected between the two groups with respect to age, sex, marital status, length of stay, and level of LTC service; however, the eating independent group had more comorbidities than the dependent group (*t* = 2.793, *p* < .006), significantly higher cognition (*t* = 4.108, *p* < .001) and physical capability (*t* = 5.258, *p* < .001), and stronger grip strength (*t* = 2.887, *p* = .005) (Table 1).

### Factors associated with independent eating performance

The results of the logistic regression analysis examining factors associated with independent eating performance are shown in Table 2. Comorbidities, cognitive function, physical capability, and grip strength were included as independent variables based on the literature review and bivariate analysis. Comorbidities and physical capability were selected as determinants for independent eating performance (Odds Ratio [OR] = 1.969, *p* = .014; OR = 1.324, *p* < .001). We estimated OR adjusted for the other variables’ effect in the model. The logistic regression analysis was significant (χ^2^ = 42.72, *p* < .001), and the explanatory power for the dependent variable was R^2^ = 42.9%. Correct classification rate was 90% for independent eating performance, 45.9% for dependent-eating performance, and 76.1% overall.

**Table 2:** Factors of the Independent Eating Performance (*N* = 117)

Variables	OR	*P*	95% CI
Comorbidities	1.969	.014	1.150–3.370
Physical capability	1.324	<.001	1.142–1.536
Cognitive function	1.062	.270	0.954–1.182
Grip strength	1.050	.286	0.960–1.149

Foot note: we estimated OR adjusted for the other variables’ effect in the model

## Discussion

This study identified significant intrapersonal factors affecting independent eating performance in OAWDs living in LTC facilities in South Korea. Significant factors identified included physical capability and comorbidities. Impaired physical capability, defined as an individual’s capacity to undertake physical tasks of daily living, may result in a more dependent life for older people [29]; retrospective study has found that functional dependence is likely to increase the proportional OR by 4.36 times, resulting in increased dependence in self-feeding [30]. Physical function with regard to moving, bending both arms, and balancing utensils is necessary for eating independently; a decline in older people’s physical capability can increase their reliance on dependent eating and, subsequently, their risk for poor nutrition. Greater efforts are needed to prevent or reduce decline in the physical capability of OAWDs in LTC facilities to support and prolong residents’ self-feeding ability [13]. A previous study [13] found that function-focused care was not effective in maintaining or increasing eating performance in OAWD living in LTC facilities; however, continuous evaluation of physical assessment and care plans tailored to physical capabilities may contribute to maintaining the physical capabilities of OAWDs. Studies have shown that integrative group exercise programs such as mindful body awareness, enhanced breathing, and aerobic exercises are effective in preventing loss of independence in OAWDs in LTC setting [31, 32].

In this study, we showed that a greater number of comorbidities significantly affected independent eating behavior. However, a study by Slaughter et al. reported that OAWDs in LTC settings with a greater Charlson comorbidity score are more prone to eating disabilities compared with OAWDs with fewer comorbidities [15], and that treating the illness may result in increased function. The reason for discrepancies in their results and ours may be due the construction of questionnaires on comorbidities. In the questionnaire used in our study, we included fewer chronic diseases, such as Parkinson’s disease, stroke, diabetes mellitus, and hypertension. Conversely, the study by Slaughter et al. [15] applied the Charlson’s comorbidity index [15], which represents the intensity of the disease [33]. Other findings suggest that the impact of comorbidities with dementia on independent eating needs further examination.

In this study, 54.7% of the participants had mild dementia and 35.9% had severe dementia. Approximately 32% of the participants were reliant on dependent eating. Also, cognitive function was not a significant factor of independent eating performance, a finding not supported by previous studies [6, 30]; however, cognitive function was significantly different between independent and dependent-eating groups, with the dependent group having lower cognitive function. Hence, there is a need for further research to examine the impact of cognitive status on independent eating performance.

In the current study, grip strength was not identified as a significant factor associated with eating performance. In a previous study, however, older people with healthy eating habits had stronger grip strength, and the grip strength of participants who ate independently was significantly stronger than that of those who ate dependently [34]. The discrepancy of results between studies may be explained by the different methods or equipment used to measure grip strength. Age-related decreases in muscle mass and strength can result in impaired function and physical capability, leading to increased morbidity and risk for mortality [35, 36]. Grip strength provides a simple, common, and reliable measure of muscle strength [37]. Future studies should consider developing different methods of testing muscle strength in OAWD according to their levels of cognition and function. Although grip strength is necessary for eating, OAWDs may also need the capability of overall body movement and coordination to eat independently.

Our findings show that depression and BPSD symptoms were not associated with eating performance. These nonsignificant findings result from the generally low levels of depression and BPSD found in our study sample. About one-fifth of the sampled participants were depressed; the mean BPSD score was 5.72 over 36, although 89.1% of the participants had one or more symptoms of BPSD. The findings indicating that eating performance and psychological factors are not associated may be due to the fact that dependent eating could be affected more by physical function than by psychological factors; however, the evidence on this is limited. More research is needed, with larger sample sizes and random sampling.

In our study, participants with independent eating performance showed increased physical capabilities and fewer comorbidities; however, OAWDs have individual patterns of life-history, health conditions, and/or cultural factors that affect eating performance [38]. Research has demonstrated that when residents are provided with person-centered eating care focused on these factors, not only does their dependent-eating behavior diminish, but their ADLs are better maintained [39]. Older adults with dementia in LTC facilities require highly specific environmental stimulation during meals, tailored to individual health conditions and characteristics [11]. Direct care workers helping patients by facilitating eating should consider each individual’s physical capabilities, comorbidities, and lifestyle including eating patterns, habits, and preferences.

The limitations of this study include its sole focus on intrapersonal level factors based on residents’ physical, cognitive, and psychosocial status; it did not include interpersonal, environmental, or policy factors. Future studies are needed to investigate factors not included in this study (e.g., characteristics of direct care workers, size/environment of facilities, staffing). As this was a cross-sectional study, we acknowledge the limitations to our conclusions about the relationship between variables and the generalizability of our results. The measurement of eating performance should be modified for more accurate assessment as we used only one item (“feeding”) from the ADL measurement. Although our study included 117 older adults living in seven LTC facilities, the sample size was relatively limited and may not be generalizable to other populations.

## Conclusions

This study investigated and provided additional information about eating performance and related factors in OAWDs in Korean LTC facilities, a subject area that has not been well explored previously. This study confirmed that physical capability is a key factor in improving eating performance in OAWDs in LTC facilities. Effective interventions to enhance eating performance should be developed, including efforts for physical capability improvement.

## Data Availability

The data that support the findings of this study are available on request from the corresponding author. The data are not publicly available due to privacy and consent considerations.

## Abbreviations

ADLs: Activities of daily living
BPSD: Behavioral Psychological Symptoms of Dementia
CSDD: Cornell Scale for Depression in Dementia
K-ADL: Korean Activities of Daily Living
K-MMSE: Korean Mini Mental State Examination
LTC: Long-term care
NPI-Q: Neuropsychiatric Inventory-Questionnaire
OAWDs: Older Adults with Dementia
OR: Odds Ratio
PCS: Physical Capability Scale
